# Key Factors Associated with Adherence to Physical Exercise in Patients with Chronic Diseases and Older Adults: An Umbrella Review

**DOI:** 10.3390/ijerph18042023

**Published:** 2021-02-19

**Authors:** Daniel Collado-Mateo, Ana Myriam Lavín-Pérez, Cecilia Peñacoba, Juan Del Coso, Marta Leyton-Román, Antonio Luque-Casado, Pablo Gasque, Miguel Ángel Fernández-del-Olmo, Diana Amado-Alonso

**Affiliations:** 1 Centre for Sport Studies, Rey Juan Carlos University, Fuenlabrada, 28943 Madrid, Spain; daniel.collado@urjc.es (D.C.-M.); juan.delcoso@urjc.es (J.D.C.); marta.leyton@urjc.es (M.L.-R.); antonio.luque@urjc.es (A.L.-C.); miguel.delolmo@urjc.es (M.Á.F.-d.-O.); diana.amado@urjc.es (D.A.-A.); 2 GO fitLAB, Ingesport, 28003 Madrid, Spain; 3 Department of Psychology, Rey Juan Carlos University, Alcorcón, 28922 Madrid, Spain; cecilia.penacoba@urjc.es; 4 Department of Physical Education, Sport and Human Motricity, Autónoma Univesity, Ciudad Universitaria de Cantoblanco, 28049 Madrid, Spain; pablo.gasque@uam.es

**Keywords:** barriers, facilitators, physical activity, lifestyle, cancer, cardiovascular disease, elderly, musculoskeletal disorders, obesity

## Abstract

Physical inactivity is a major concern and poor adherence to exercise programs is often reported. The aim of this paper was to systematically review published reviews on the study of adherence to physical exercise in chronic patients and older adults and to identify those adherence-related key factors more frequently suggested by reviews for that population. The Preferred Reporting Items for Systematic Reviews and Meta-Analyses (PRISMA) guidelines were followed. Results were classified considering the target population and participants’ characteristics to identify the most repeated factors obtained for each condition. Fifty-five articles were finally included. Fourteen key factors were identified as relevant to increase adherence to physical exercise by at least ten reviews: (a) characteristics of the exercise program, (b) involvement of professionals from different disciplines, (c) supervision, (d) technology, (e) initial exploration of participant’s characteristics, barriers, and facilitators, (f) participants education, adequate expectations and knowledge about risks and benefits, (g) enjoyment and absence of unpleasant experiences, (h) integration in daily living, (i) social support and relatedness, (j) communication and feedback, (k) available progress information and monitoring, (l) self-efficacy and competence, (m) participant’s active role and (n) goal setting. Therefore, adherence to physical exercise is affected by several variables that can be controlled and modified by researchers and professionals.

## 1. Introduction

The increase in life expectancy and the remarkable advances in medicine have caused the mean age of the global population to progressively increase [1]. Linked to this, the number of people suffering from one or more chronic diseases has dramatically risen, which leads to large economic costs for countries and a greater need for health care services [2]. Physical exercise has become standard practice in clinical care due to the fact that it leads to numerous benefits in many different pathological and non-pathological populations [3]. In this regard, the inclusion of physical exercise programs in treatment plans is now an essential tool for most health care providers, while the use of evidence-based exercise programs subjected to the evaluation of exercise professionals is considered key in the prevention and treatment of many conditions [3,4]. 

Every year, hundreds of randomized controlled trials aimed to evaluate the effects of physical exercise on health-related variables are published. That is the consequence of a large investment of public and private institutions and the work of many researchers, who have demonstrated that physical exercise leads to numerous benefits in many different pathological and non-pathological populations [3]. As a result, physical exercise has become a medicine that everyone should take regularly [5] and many different campaigns, advertisements, and policies have been developed to spread that message [6,7]. However, although most people know the relevance of having an active life as a part of a healthy lifestyle, inactivity is still a major concern and the number of sedentary people has not been properly reduced [8,9]. 

Therefore, there is a key question that researchers working on physical exercise and health must ask: why people not exercise even knowing how good it is for their own health? The answer to this question may be related to the lack of proper information that enhances motivation and adherence to the exercise guidelines proposed. For example, the World Health Organization (WHO) has recently published new guidelines on physical activity and sedentary behavior, indicating that adults should do at least 150–300 min of moderate-intensity aerobic physical activity, or at least 75–150 min of vigorous intensity aerobic physical activity, or a combination of moderate- and vigorous-intensity activity throughout the week, in order to obtain substantial health benefits [5]. However, these guidelines lack any information regarding strategies to ensure that individuals maintain these levels of physical exercise over time nor strategies to motivate sedentary individuals to start exercising. Hence, it seems that neither the knowledge of the benefits of exercise nor the setting of minimum thresholds of exercise are driving forces to reduce sedentarism effectively. 

Low levels of adherence to exercise may cause some randomized controlled trials aimed to assess the benefits of exercise on one or several health outcomes to not achieve significant results [10,11]. However, the adherence problem may be bigger when talking about the general population that does not participate in those kinds of studies since participants in randomized controlled trials are usually volunteers, selected according to inclusion and exclusion criteria that often rejects individuals with severe impairments that may reduce their adherence (for instance, it is usual that people with severe cognitive impairment are excluded in articles involving older adults or Parkinson’s Disease patients [12,13] and people with bone metastasis are excluded in cancer studies [14]). Furthermore, as volunteers, it can be assumed that participants in studies may be inherently motivated, or at least they are willing to be involved. However, this cannot be assumed for everyone in the general population and it could happen that patients engaging in clinical trials may not be representative of the population in question, since their psychological predisposition to exercise may be different than the predisposition of the others [15]. In addition to the selection of volunteers and ideal participants, randomized controlled trials often tend to be highly supervised, which may explain why the levels of adherence are often higher compared to observational studies [16].

Many theories and models have been proposed from different disciplines to explain the “adherence to exercise” phenomenon [17,18]. Some articles have suggested the need for a parallel psychological intervention, in addition to the exercise program, to aid in behavior change [19]. Others have proposed the benefits of increasing participants’ motivation towards exercise by paying more attention to the three basic psychological needs: autonomy, competence, and relatedness [20]. Previous studies have also tried to increase the adherence to physical exercise programs by including technological gadgets or by proposing alternative forms of exercise [21,22]. There are even some authors who have suggested the convenience of paying people for doing exercise, as some insurance companies have started to do, knowing that their clients will be healthier if they are physically active and their incomes will be accordingly increased [23]. 

Despite the large number of studies on adherence to exercise, the very concept of adherence is not well established and varies from one study to another, confusing adherence with other terms like attendance, i.e., the number or the percentage of sessions attended [24]. Another not fully appropriate way to conceive adherence to exercise is by counting the number of dropouts during their exercise intervention. Following this last conception, one could interpret good adherence to their program when the percentage of their participants who finished the intervention is high. In this regard, the Physiotherapy Evidence Database (PEDro) Scale suggests that measurements of the key variables should be obtained from more than 85% of the initially randomized participants [25]. Thus, that criterion is based on the number of dropouts, but it does not establish the need of completing a minimum percentage of sessions (attendance) nor the involvement of the participants during the sessions. In this regard, adherence has been described as the extent to which the behavior of a person correlates with the agreed plan of the suggested exercise intervention, so it would be related to the degree to which the target intensity and volume are achieved [24,26]. Therefore, adherence to exercise is a concept with deeper roots in the participant’s behavior than a mere number of dropouts or percentage of sessions attended.

Following this last definition of adherence that includes the assessment of the intensity and volume achieved, new advances in technology have made easier the assessment of adherence given that the exercise intensity and volume can be more easily monitored or even self-monitored by the participant [27]. However, the use of technology involves other problems that should be considered, such as economic costs, the additional time required to set it, the potential unpleasant or uncomfortable experiences, the difficulties experienced by the participants to use it appropriately, and the shame or simply the reluctance of people who do not want to use it [21,28].

Despite all the above-mentioned information and the number of articles published on this topic, to date, there are mixed and inconclusive results regarding the best practices for increasing exercise adherence, with large heterogeneity in terms of physical exercise type recommended, the psychological approach used, the target population, the need to treat a chronic disease, the age of patients and the main aim of the exercise interventions. Therefore, the current study aimed to systematically review published reviews on the study of adherence to physical exercise in chronic patients and older adults and to identify those adherence-related key factors more frequently suggested by reviews for chronic disease patients and older adults.

## 2. Materials and Methods

The current systematic review of reviews has been developed following the Preferred Reporting Items for Systematic Reviews and Meta-Analyses (PRISMA) guidelines [29].

### 2.1. Search Strategy and Selection of Studies

The search for published studies was conducted in October 2019 in the scientific databases PubMed (MEDLINE) and Web of Sciences (including KCI-Korean Journal Database, MEDLINE, Russian Science Citation Index, and SciELO Citation Index). The terms used for the search were “adherence”, “exercise”, and “systematic review OR meta-analysis” separated by the Boolean operator AND. To select articles focused on adherence to exercise and avoid those that only included adherence as a secondary or complementary measure, the term “adherence” had to be within the title of the article. Only articles published in the last 10 years (2010 to present) were included in the search to show an updated picture of the topic. The search for published studies was independently performed by two authors (DC-M and AML-P) and disagreements were resolved through discussion.

Screening of searched articles and its subsequently full-text review was carried out regarding the following inclusion criteria: (a) systematic/narrative review and/or meta-analysis design, (b) studies focused on patients with chronic disease patients or older people as the target population, (c) focused on any type of physical exercise and (d) aimed to identify factors associated with adherence to exercise. Besides, articles fulfilling the following criteria were excluded: (a) reviews written in any language different from English or Spanish, (b) studies focused on the concept or definition of adherence, (c) articles aimed to analyze the relationship between adherence and intervention’s effects, (d) reviews focused on the methods used to assess adherence. 

### 2.2. Data Extraction

The identified review articles were distributed among all the authors of this study and data extraction was performed by duplicate. For each article, the researcher manually extracted the information about the population, intervention, aim, conclusion, and study design, following the PICOS (Population/Problem, Intervention, Comparator, Outcome and Study Design) approach. Key factors reported in reviews and meta-analysis were extracted by checking the results, discussion, and conclusion sections of each article. These key aspects were factors identified in the reviews that may affect adherence to exercise in the target populations. After this, the information obtained through data extraction was compared between the two researchers assigned to each article, and all observed differences were scrutinized and corrected. A third researcher was sought in the case of discrepancy.

### 2.3. Data Synthesis

After extracting the data, another author checked and combined the information of each article and prepared the tables that summarize the data of all articles (Supplementary data Table S1). The main key factors extracted from the articles were grouped in topics to enhance the comprehension of the results outcomes. This classification of findings was performed based on the identified factors from the studies included in this review and included:

1. Characteristics of the exercise program, that would comprise those factors related to how the physical exercise is planned, including the individualization, the evidence-based settings, and other characteristics such as frequency, duration, intensity, or volume.
2. Involvement of professionals from different disciplines, that would be related to the convenience of including experts or methods from different disciplines.
3. Supervision, which would include the significance or irrelevance of supervising the exercise interventions.
4. Technology, which would be focused on the potential additional benefits or disadvantages of including technological devices and applications to conduct the physical exercise intervention.
5. Initial exploration of participant’s characteristics, barriers, and facilitators, which would include the identification of relevant variables of the patients before the exercise interventions that could reduce or increase the adherence to exercise.
6. Participants’ education, adequate expectations, and knowledge about risks and benefits, which would be related to what the participants know or learn about the relevance of physical exercise for their own health so that the expectations about the improvements were not inaccurate.
7. Enjoyment and absence of unpleasant experiences, which would be related to the pleasure obtained while exercising and also by the absence of pain or discomfort.
8. Integration in daily living, which includes the consideration of the participant’s preferences and background to adapt the exercise characteristics and settings.
9. Social support and relatedness, which includes support from peers, staff, and family, as well as the establishment of positive social interactions and feelings of belonging to a group.
10. Communication and feedback, which is related to the effective interaction between the staff and the participant.
11. Available progress information and monitoring, providing enough information to the patient so that they can be aware of the changes and improvements from objective data.
12. Self-efficacy and competence, which is related to the participant’s perception of what they can do and what they will be able to do.
13. Participant’s active role, which would include self-management, self-control, self-monitoring, autonomy, and empowerment.
14. Goal setting, which is related to the establishment of adequate objectives.

Afterward, the reviews were classified considering the target population and participants’ characteristics in order to identify the most repeated factors obtained for each condition: cancer, cardiovascular disease, older people, participants with musculoskeletal pain, obesity patients, and exercise referral schemes.

## 3. Results

### 3.1. Study Selection

**Figure 1:**
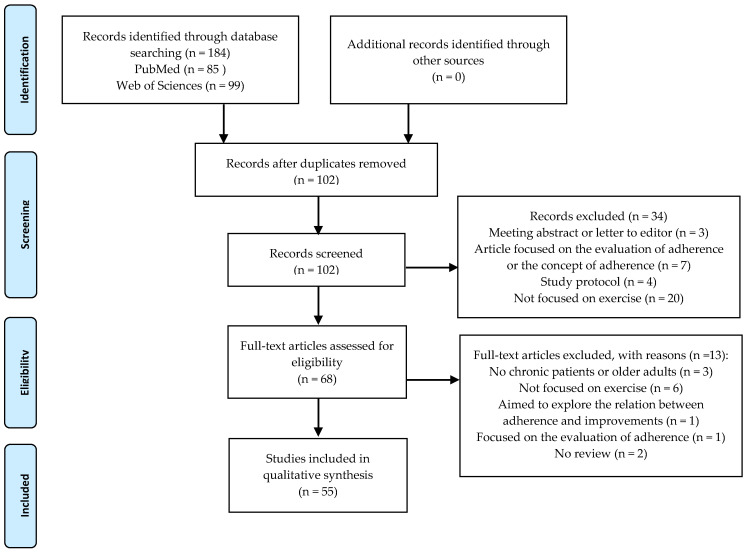
Flow diagram of the selection process.

In the original database search, 184 studies were initially identified, 85 articles in PubMed and 99 in Web of Sciences (see Figure 1). After removing 102 duplicated articles, studies were screened by analyzing their titles and abstracts. Subsequently, 34 records were excluded due to different reasons: three were abstracts or letters to the editor, seven were focused on the assessment of adherence or its concepts uniquely, four were study protocols and twenty were not focused on exercise. The full-text total of 68 articles was re-viewed and 13 of them were excluded for different reasons: not having chronic patients or older adults as target population (*n* = 3), not being focused on exercise (*n* = 6), focused on the evaluation of adherence (*n* = 1), aimed to explore the relationship between adherence and physical exercise improvements (*n* = 1) or not being a review (*n* = 2). Thus, 55 articles, published from 2010 to 2019, were finally included in the current umbrella review.

### 3.2. Study Characteristics

The main characteristics of the selected reviews are reported in Table 1. The 55 reviews included 11 meta-analyses. Regarding chronic patients and older adults, seven articles were focused on cancer [11,14,30,31,32,33,34], seven on cardiovascular disease [28,35,36,37,38,39,40], eight on elderly people [21,41,42,43,44,45,46,47], twelve on musculoskeletal disorders [48,49,50,51,52,53,54,55,56,57,58,59,60], three in obesity or weight loss [16,61,62], six on multiple chronic diseases [12,27,63,64,65], two on intermittent claudication [66,67], two on population with mild cognitive impairment and dementia [68,69], and single articles analyzed Parkinson’s disease patients [13], type-2 diabetes patients [70], solid-organ transplant candidates [15], and participants under vestibular rehabilitation [71].

Thirteen of the included revisions were based on exercise interventions conducted in public or private centers, while five studies were focused on adherence to home-based exercise programs [41,48,58,71,72]. The remaining 37 reviews incorporated exercise programs mixing center- and home-based interventions and in some cases also physical activity interventions, like walking or leisure time activities. Although all the articles compromised exercise interventions, some also included physiotherapy [52,55,58,60], lifestyle-changing interventions [33,61,62,70], exercise referral schemes [65,73,74,75], technology and multimedia effects [21,27,38,64], behavior change techniques [47,49,50,56,62], and barriers and facilitators to exercise [46,52,59,69,75]. Those studies were included since they provided valuable information about factors associated with adherence to exercise.

### 3.3. Outcome Results

The analysis performed revealed 14 key factors of exercise programs that may positively influence their adherence rates. As Table 2 shows, these topics reported different sub-key aspects that represent in more detail the characteristics of the most adhered programs. 

First, the results of the global analysis revealed that initial exploration of participant’s characteristics, barriers, and facilitators seemed to be crucial to enhance exercise adherence in general chronic patients and older adults. Concretely, thirty-six reviews identified the importance of pre-participation evaluation of participants’ previous lifestyle habits as well as their physical and mental health status. Besides, 29 reviews stated that possible barriers and facilitators to exercise may need to be contemplated before the program’s delivery. The next most distinguished key aspect, mentioned by twenty-nine articles, was to study participants’ preferences and backgrounds to enhance the integration of exercise in their lifestyle. Moreover, regarding the program design characteristics, twenty-three reviews stated that developing an individualized exercise intervention could be a key point to enhance adherence rates. Although the psychological variables did not reach such high support in the general analysis, it appeared that fomenting participants’ self-efficacy may be the most useful psychological factor in exercise adherence (21 articles).

As for the individual analysis of different health conditions registered, also presented in Table 2, considering patients’ previous habits and physical and mental health status was the most valuable aspect in patients with cancer [11,14,30,31,32,33,34], patients with cardiovascular disease [28,35,36,37,39,40], patients with musculoskeletal disorders [48,49,52,54,59] and in exercise programs aimed to reduce obesity or in weight loss exercise interventions [16,61,62]. In older adults, five reviews supported the provision of objective information about their progress and the consideration of participants’ preferences and background as effective strategies to enhance adherence [21,41,42,46,47]. However, supervision by a health care or exercise professional, individualization of the exercise program, providing information about exercise risks and benefits, and the election of an accessible location for the development of the exercise program were each supported by four out of eight reviews. 

Outlining briefly each of the chronic diseases, cancer exercise programs may also need to analyze patients’ barriers and facilitators to exercise before the intervention [14,30,31,33,34] and choose good accessibility and an adequate place to deliver the intervention [11,14,30,34]. Exercise programs for patients with cardiovascular disease should analyze exercise barriers and facilitators before the intervention [28,35,36,37,39,40], but also, they should emphasize the importance of family and peer support [28,36,37,38,39], together with exercise monitoring [28,35,37,38] and providing educational information about how to exercise in their condition [35,36,37,38]. When interventions were carried out by participants with musculoskeletal disorders, an assessment of the barriers and facilitators appeared to be as crucial as analyzing their preferences and background and developing multidisciplinary programs (five articles in each key aspect). Finally, in weight loss interventions, or with obese participants, patients’ preferences and backgrounds to enhance the integration of the program in their lifestyle seemed to be essential [16,61,62].

## 4. Discussion

The main aim of the current systematic review of reviews was to identify key factors associated with adherence to physical exercise in patients with chronic diseases and older adults. Many different key factors were identified in the included reviews as positive to promote adherence to exercise and they were organized and summarized in the following lines.

### 4.1. Design of the Exercise Intervention

Regarding the design of the exercise program, two main key factors were identified: (a) the individualization and the scientific basis of exercise type and (b) the duration of the exercise program in weeks. The first key aspect can be divided in two since 21 of the included reviews found that tailored exercise is necessary to achieve high levels of adherence, and seven identified the need of conducting exercise interventions with a scientific background, with five of those seven arguing that both aspects are relevant. The other key factor identified by 10 reviews was the duration of the exercise. It was shown that longer exercise interventions were related to lower adherence to the program. This outcome may be associated with the need to maintain a homogeneous exercise routine during the entire exercise program in randomized controlled trials, which may cause some individuals to drop the program due to the lack of variety. In this regard, the measurement of adherence in programs that allow the change of exercise activities across the program may be necessary to determine if the constraints of randomized controlled trials may be overcome by affording patients more liberty to decide the exercise type. 

From a patient-centered perspective, the individualization of the exercise in terms of type, intensity, duration, frequency, but also in needs and interests, is necessary for effective promotion of adherence. This would elicit a superior response not only due to a better adjustment to the physiological demands of the activity but also due to enhanced patient perception towards the exercise program. For instance, among patients with dementia, those with better cognitive health often have lower adherence rates [69]. This could be related to a non-adequate adjustment of demands, which may be only tailored to those who have the poorest cognitive capacity in the group. That could also be true for groups comprised of patients with different functional capacity, since exercise may be tailored to those with the poorest physical function. Therefore, making homogeneous groups in terms of interests, needs, and functionality will increase the adjustment of exercise demands, social support, connectedness, and relatedness [42] as well as achieve a superior physiological and psychological response.

Although some authors have pointed that the characteristics of the exercise program may be related to exercise adherence, some aspects, like the type of exercise or exercise intensity, are not often reported as key factors to promote adherence. In this regard, we found a similar number of reviews showing that variables like intensity, frequency, or volume are relevant and reviews reporting that they are not. Traditional exercise interventions such as walking may reduce adherence compared to alternative options, such as Nordic walking, resistance training, or circuit training [67], but walking can also be considered as an accessible and feasible form of exercise that facilitates the attractiveness of the exercise program for some individuals [46]. Regarding the exercise frequency, it has been shown that one single session each week may lead to lower adherence, probably due to participants doubting the efficacy, the less frequent contact with the staff and peers, and the bias caused by the selection of physically active participants who may be unsatisfied with the low exercise frequency [45]. Furthermore, if participants only do exercise within the exercise program, they would not be following the recommendations of the WHO [5].

Findings related to the duration of the exercise program are extremely alarming. Ten reviews showed that the longer the duration of the intervention the lower the adherence obtained in the individuals that underwent the program. With the aim of increasing the long-term adherence to physical exercise, it seems that there is a need for alternatives to escape from routine and avoid interventions that could bore or overwhelm the patients [45]. This finding may conflict with scientific aims. Since it is known that certain variables may need a couple of months to be improved by physical exercise, reducing the duration of the interventions may not be an adequate alternative. So, when a specific intervention length is required, researchers and physical exercise professionals must make an effort to facilitate the accommodation of exercises within the daily living of patients [54]. 

In sum, individualization, the use of various exercise types with proven evidence of efficacy for the target population, a frequency higher than once per week, and a moderate duration of the program may be key factors to promote exercise adherence.

### 4.2. Multidisciplinary Team

A total of 12 reviews identified how the presence of different professionals who conduct the exercise intervention could improve adherence. In addition to the physical exercise professional, who is mainly responsible for the design and development of the exercise program, the addition of counseling by other professionals such as psychologists, physicians, physiotherapists, nutritionists, or nurses is habitually perceived as positive to reinforce adherence. In this regard, some reviews [12,41,47,65] showed that the participation of physicians was key since patients were more likely to adhere to physical exercise when it was prescribed by a healthcare professional. Furthermore, the labor of psychologists may enhance adherence to exercise by conducting different behavioral change techniques or cognitive-behavioral programs [49,50,55,56,60,65]. However, the efficacy of these programs is still controversial, obtaining mixed results and large heterogeneity.

Therefore, the presence of a multidisciplinary team may contribute to increased adherence to exercise among chronic patients and older adults. Although the presence of different specialists may be affordable in controlled trials and other research designs, the costs of the exercise program may be largely increased by this factor, which could impact the price that the user must afford to be involved in the program and consequently the adherence of exercise, especially for those with at a low socioeconomic level [69]. This brings us to another relevant issue: the willingness to pay for exercise. A recent study showed that only half of the older adults were willing to pay for fall prevention programs [76], while chronic patients suffering from knee osteoarthritis may be willing to pay little money, with only 26% willing to pay more than €65 for six weeks of an evidence-based program and only 10% willing to pay more than €100 [77]. Although the benefits of exercise are well-known, people are still reluctant to pay for it, even a low amount of money. Taking this into account, it is challenging to provide an exercise program involving professionals from different disciplines that suits the low willingness to pay of older adults and chronic patients. 

In sum, the addition of professionals from different disciplines such as psychologists, physicians, or nurses may increase adherence to physical exercise interventions.

### 4.3. Supervision during the Exercise Sessions

Supervision was identified as a key aspect by 17 reviews. Supervision during the exercise session involves at least one professional checking how the participant is performing the prescribed exercises, which indeed enhances the quality of the execution and, consequently, increases the potential benefits and reduces the possible risks of inadequate executions [16,44,66]. Furthermore, supervision also makes the evaluation of adherence easier and more accurate, avoiding the use of self-reported exercise registries and problems related to the use of monitoring technology. In this regard, Hughes, Salmon, Galvin, Casey, and Clifford [44] showed that adherence (assessed through self-reported registries) to exercise was higher in unsupervised home exercise programs while the benefits were lower than those observed in class-based supervised exercise. This could be explained by an over-estimation of adherence rates due to social desirability and obsequious responses from the participants [12]. However, the advantages of supervision are documented, allowing participants to access the professional’s knowledge, feedback, and support, which may increase self-efficacy and reduce the discouraging feeling and potential risks [16].

The debate between face-to-face vs. home-based exercise is not associated with supervision, since both types of delivery can be supervised, provided that the professional can be monitoring what the participants are doing during sessions in both types of exercise programs. As a disadvantage of supervised exercise, the costs of the programs may be increased and the flexibility in time can be reduced since both participants and professionals must be simultaneously in the same place or connected online, which could reduce the adherence as a consequence of incompatibility in timetables.

In sum, exercise programs supervised by at least one physical exercise professional may increase adherence to physical exercise. However, other disadvantages have been described.

### 4.4. The Use of Technology

A total of 12 reviews have analyzed the role of technology in enhancing adherence to physical exercise. Although promising results have been achieved in technology-based exercise programs like those obtained by Xu, Li, Zhou, Li, Hong, and Tong [38], who observed that the completion was 1.38 times higher compared to traditional programs, the evidence is still debated [27]. Some advantages have been reported, since the technology may be useful to accurately monitor the physical activity of participants in terms of frequency duration and time, individualize the exercise prescription, provide real-time feedback, to make reminders, connect professionals and patients, share the performed activities with peers, and provide instructions, among other benefits [44,64,71]. 

However, in chronic patients and older adults, technology must be used with caution, since not everybody will have the same response. Older adults may be less likely to engage in physical exercise technology-based programs while young people may have the opposite perception [28]. However, another review [21] found that dropout rates were similar in technology-based and traditional exercise programs, whereas the reasons were different. In this regard, reasons to abandon traditional exercise were lack of motivation and personal obligations, while in technology-based programs reasons included low motivation, lack of interest, discomfort, lack of time, limited space at home, technology usability, or shame. Furthermore, it must be noted that when the exercise program is based on technology, the interpretation of adherence may be different, since sometimes researchers are assessing adherence to a device instead of adherence to physical exercise [28]. 

In sum, the use of technology may be recommended when participants are willing to use it and the devices and software are adequate for them, but it must be noted that some people may refuse technology. Therefore, it could be suggested that the inclusion of technology may be voluntary and not mandatory in exercise programs.

### 4.5. Initial Exploration of Participant’s Characteristics, Barriers, and Facilitators

More than 70% of the included reviews identified the need for carrying out a pre-participation comprehensive analysis of the participants’ characteristics and the potential barriers and facilitators. Thirty-six of the 55 included reviews found that some aspects like the health status (including physical and mental health) or previous habits (such as physical activity level, smoking, or alcohol intake) are relevant factors to predict exercise adherence. Without any doubt, these factors are the most widely cited in the scientific literature about adherence to exercise in chronic patients and older adults. 

Health status stands out as one of the major factors for exercising. This is a complex concept that needs to be understood not only in terms of severity of symptoms, but also in terms of health-related physical function and other components like mental, cognitive, social, or sexual status. Depending on the condition, different health-related aspects will be linked to lower adherence. In this regard, chronic diseases that involve pain or fatigue may reduce the attendance and adherence of patients [78,79]. Furthermore, those patients with depression will also be more likely to abandon the exercise program [43,72,80]. On the other hand, no difference has been observed when comparing the adherence rates of patients with conditions like cancer, cardiovascular disease, and diabetes [63]. Although therapists cannot modify the baseline health status, it is relevant to properly assess and analyze the status before the exercise program is conducted. Furthermore, barriers and facilitators must be openly discussed before and at regular intervals throughout in order to ensure individualization [61].

Within the suggested initial exploration of barriers and facilitators, the stage of change is a strong predictor of adherence to exercise in chronic patients [31]. The concept of stage of change comes from the transtheoretical model, which defines a series of behavior change stages: pre-contemplation, contemplation, preparation, action, and maintenance [81]. In this regard, a mismatch between the stage of change and the selected strategy may lead to lower adherence rates and those patients who are not physically active are more likely to report exercise barriers, but the sedentary behavior does not necessarily hinder patients from becoming physically active [31]. 

The diagnosis of a chronic disease may involve several changes in the daily living of patients, reduction in physical activity levels being one of the major changes. Among others, the diagnosis of Inflammatory Bowel Disease has been related to a reduction in physical activity levels [82], and even those patients with high pre-illness physical activity levels drastically increase their sedentary behavior while undergoing cancer treatment [83], especially when they had previous sedentary habits [32,34]. On the other hand, some middle-aged and elderly people may start to exercise after they are diagnosed with a chronic disease [84]. Other changes have been reported after hypertension diagnosis, such as smoking cessation accompanied by a small reduction in inactivity [85], but changes in lifestyle are often not enough. Although the implications of diagnosis are still controversial, it is clear that it is a time where people can be more prone to change, since it could be interpreted as a “wake-up call” to adopt a more healthy lifestyle even when that intention is not translated into real changes [86,87]. This is similar to the concept of “teachable moments”, which has been defined as opportunities for changing unhealthy habits after a specific circumstance or event [88]. These moments may be a diagnosis, a hospitalization, or other episodes, and are influenced by all the involved healthcare professionals and require communication skills [65].

Apart from the potential internal barriers, there are other contextual and cultural barriers that should be considered. For instance, socioeconomic status is a common barrier [35,43,69] and women in some cultures may feel uncomfortable walking unaccompanied or simply being physically active [46]. Therefore, social and economic factors, as well as beliefs and group norms, must be considered when an exercise program is designed.

In sum, a comprehensive baseline assessment must be carried out before the exercise program to identify potential barriers and facilitators, including health status (physical and mental health) and previous lifestyle habits.

### 4.6. Participants Education, Adequate Expectations, and Knowledge about Risks and Benefits

This is a relevant issue that included the education in exercise and health (17 reviews), adequate information about benefits and potential risks (15 reviews), and adequate expectations of changes (15 reviews). In general terms, it can be said that those patients who are aware of what exercise can do for them are more likely to adhere to exercise programs. Although in the last years there have been a lot of campaigns trying to disclose the benefits of being physically active, additional effort must be expended to educate people on exercise and health.

People often show higher levels of adherence when the exercise is prescribed by physicians rather than by other professionals. That is in line with the health belief model, which states that the expected benefits are key to be involved in an activity [89]. Thus, when participants believe that their health status is going to be enhanced, they are more likely to be involved in the exercise. In fact, this factor could partially explain why some physical exercise programs achieved better adherence than others. For instance, in fall prevention interventions, the adherence to programs aimed to improve balance was higher than adherence to programs aimed to increase flexibility [41,45]. That could be explained by the participants’ expected benefits, who may believe that flexibility exercise is not going to lead to substantial improvements that lead to the prevention of a fall episode. Therefore, patients must always be adequately informed about the objectives and the expectations of each exercise.

However, too high expectations may be a double-edged sword. One of the included reviews analyzed the effects of expectations on adherence to a weight loss program. They observed that those individuals with lower expectations had better adherence, while those with unrealistic goals were more likely to abandon the program [61]. This was also observed in exercise referral schemes [73]. Therefore, physical exercise professionals must be cautious when they discuss goals and expectations with the participants in order to avoid unrealistic or overly optimistic beliefs. Furthermore, it has been suggested that these expectations should be based on health and quality of life variables, and not just measures like weight loss in a specific time length.

In line with the avoidance of overly optimistic expectations, potential risks must be ethically disclosed, and the presence of unpleasant feelings should be anticipated [36]. They must also be advised about the progress of their disease and what the absence of exercise may cause. For instance, it has been shown that colorectal adenoma patients may be unaware of the increased cancer risk they have, [33] and patients with heart failure had poor knowledge about their disease [90] and this could reduce the engagement in healthy activities. 

In sum, participants in exercise programs should be educated in order to be aware of the health benefits of exercise and the risks of sedentary habits. They should also be adequately informed about the usual feelings during the practice (for example the fatigue) and must be provided with enough information to have realistic expectations of change, avoiding overly high or low expectations.

### 4.7. Enjoyment and Absence of Unpleasant Experiences

The experience of participants while doing exercise is crucial to enhance the adherence to physical exercise. Enjoyment is an immediate reward that could lead to better persistence than delayed rewards, such as health benefits in the long-term [91]. It has been related to participation and efficacy of physical exercise [92] and is closely associated with intrinsic motivation [93]. In this regard, participants are more likely to enjoy the practice when the basic psychological needs of competence, relatedness, and autonomy are satisfied [94]. Those needs have also been identified in this systematic review as key factors to be considered. The use of adequate technology may also enhance the enjoyment in chronic patients [95] and alter the perceived effort of patients doing exercise, achieving similar physiological responses with lower perceived exertion [96].

On the other hand, the presence of unpleasant experiences may limit participation and adherence to exercise. This is especially relevant among those patients suffering from conditions that cause pain or fatigue and can be increased when the intensity of exercise has risen [97]. This is consistent with the notion that enjoyment is a strong mediator of adherence to exercise in patients suffering from musculoskeletal pain [98].

The affective response to exercise is based on the interpretation of a complex net of interactions among physiological demands, participant’s psychological characteristics, the environment, and situational appraisals [99]. Therefore, these individual interpretations will be different from subject to subject, so what is enjoyable for someone could lead to unpleasant feelings in others. For instance, some people can interpret the increased heart rate, breathing, or temperature as pleasant or not [100]. Similarly, physical tiredness could be unpleasant but also lead to emotionally pleasant feelings [100]. In the case of pain, clinicians must adequately inform about pain experience and beliefs, reducing the associated fear or anxiety and avoiding the immediate abandoning of activities that generate a little discomfort [52].

In the case of obesity, other unpleasant feelings can emerge, such as the embarrassment with their own appearance while doing exercise, rapid exhaustion related to poor physical conditioning due to physical inactivity, or lack of movement enjoyment [61,101]. 

In the search process, this systematic review of reviews also identified reviews aimed to evaluate the effects of paying people to exercise. Mitchell, et al. [102] found that financial incentives increase attendance in interventions for up to six months. This approach is controversial since, according to the self-determination theory, giving external motivators in activities that could be intrinsically enjoyable may reduce the intrinsic motivation once the external reward is removed [103]. However, this harm to intrinsic motivation may be lower among previously inactive patients who lack intrinsic motivation to exercise [23]. Therefore, financial incentives may be only adequate in those individuals that would not exercise under any other conditions.

In sum, the pleasant and unpleasant feelings during exercise are going to affect adherence and motivation. Enjoyment is an immediate reward that may increase adherence more than other delayed rewards such as health benefits. Furthermore, unpleasant experiences during the exercise practice are also common in patients with different conditions, so it is necessary to give enough information so that the patients adequately interpret their feelings and emotions and reduce the associated fear, anxiety, and avoidance.

### 4.8. Integration in Daily Living

One of the keys that was reported by more than half of the reviews is the integration of physical exercise in daily living. This is a factor that may be affected by the rest of the identified factors and involve the transformation of physical exercise into a lifestyle habit, which would avoid the common barrier associated with the perceived lack of time [14,59,61]. Many variables can affect that process, such as the flexibility at work [104] or the intimidating gym environment [75], but the background and preferences of patients are the most crucial ones. In this regard, good accessibility (in terms of money, distance from home, physical barriers, etc.), as well as relative flexibility in the timetable (for instance including the possibility of making up the sessions that patients were not able to attend within the week) have been identified by 21 reviews. Only exercise programs that are in line with the preferences and characteristics of participants can become an actual habit.

In the scientific literature, many comparisons between center-based and home-based exercises can be found. The first type could be more effective since a professional is often supervising and controlling the execution. Furthermore, the presence of a professional can also lead to higher levels of adherence due to the social support, feedback, and relationship between the staff and the patient, as previously mentioned [34]. However, it has been suggested that in the long-term, people could be more likely to adhere to home-based programs [13,105] because they are easier to integrate into their lives. In this regard, a potential explanation is that the integration of home-based exercise in daily living could be easier than the integration of center-based exercise since some barriers can be avoided when exercising at home. Thus, it still remains controversial since adherence to home-based exercise programs may be as engaging as center-based ones [30,63]. Another review found that exercising at home is perceived as more convenient by older people, reducing some barriers such as the weather conditions, the lack of transport, or the feelings of intimidation to attend a center or to be in a group [21]. This could also be applicable to cancer patients during treatment [34].

In sum, only those exercise programs which are in line with the preferences and characteristics of participants can become an actual long-term habit. In this regard, home-based exercise interventions may increase the chances of it becoming a habit, but other aspects such as the supervision by a professional must be considered in order to increase social support, feedback, and improve the relationship between staff and patient.

### 4.9. Social Support and Relatedness

The relevance of social support from staff, family, and peers is supported by 22 reviews, while 11 reviews identified “relatedness” as key. Relatedness could be defined as the feeling of belonging to a group and is one of the basic psychological needs to improve motivation according to Self-Determination Theory [20]. This is different from the concept of “social support”, which may be defined as “support accessible to an individual through social ties to other individuals, groups, and the larger community” [106]. Thus, it involves a network of people—including family, friends, and community members—who are available to provide any kind of help or support. In the context of the physical exercise, we can include the people who oversee the program, which could be limited to the technician, or also include other professionals such as the physician. In addition to adherence, previous research has shown that poorly perceived social support has been related to lower mental health, higher risk of developing certain diseases, lower life expectancy, lower physical activity levels, more stress and poorer resilience [107]. On the other hand, satisfactory social support can encourage optimism and self-esteem, reducing stress and depressive symptoms [108], which could affect adherence to exercise.

The ability of the physical exercise professional to be close and available, to create an environment where everybody is comfortable, and to make homogeneous groups that promote the positive relationship with other patients will impact both the relatedness and the social support and increase adherence to exercise [42,69]. 

Technology may be used to share the data (such as the number of steps, trails, etc.) with different people, which could increase motivation. In this regard, data obtained by mobile applications may be shared with health care providers, which could positively affect the communication and perception of support from these professionals [38,109]. Furthermore, some devices and apps may promote physical activity engagement by sharing the physical activity with app-specific communities in existing social networking platforms, which has been associated with social support [110,111,112].

In sum, positive social interactions with the staff and other participants may increase adherence to exercise. The social skills of the staff and the creation of homogeneous groups are crucial to achieving high levels of social support and relatedness.

### 4.10. Communication and Feedback

Communication and feedback from the staff may be related to social support from the physical exercise professional and health care specialists. Regular communication out of the usual timetable, such as phone calls, home visits, app-based interactions, reminders, or booster sessions may increase social support, exercise adherence, and the amount of physical exercise in the short- and long-term [38,40,47,58,71]. In the case of people suffering from cognitive impairment, communication is also essential to make reminders and provide more information [68].

The communication method may be relevant since technological and multimedia approaches may have a positive effect on adherence compared to traditional paper-based or verbal interaction [44,64]. Among potential explanations, it has been suggested that multimedia instructions may be more motivating because they can be more novel, realistic, and personalized. Thus, investment in technology may be justified as long as patients own and are familiar with technology [64].

These contacts can be used to be in touch and reinforce the behavior, as well as to identify potential non-expected barriers. Positive feedback and reinforcement of efforts have been identified as key aspects to enhance motivation [57], increasing the probability of the repetition of desirable behavior by the association between the response and the stimulus [113]. This type of feedback is different from the results-based feedback, which would be based on the effects of the exercise and the results achieved (weight loss, blood pressure reduction, pain reduction, etc.). Other types of feedback, such as the number of kilometers walked, or the estimated number of calories expended, are also directly related to the effort of the participant but will be discussed in the following subheading.

In sum, communication and feedback from the staff, including not only the interactions during the sessions but also the regular communication out of the usual timetable, may increase social support, exercise adherence, and the amount of physical exercise in the short and long term.

### 4.11. Available Progress Information and Monitoring

The feedback on outcomes is also an important variable that has been identified as key factor for adherence in 17 reviews. Several studies have identified that the perceived benefits are crucial to continuing exercise. On the other hand, in some conditions like advanced cancer, the progression of the disease seems to be the main cause of abandoning the exercise program [14]. Sometimes, the subjective perception of the benefits will be enough to motivate people to continue with exercise. However, the inclusion of objective measures that the patient can understand and interpret will support those perceptions. In this regard, a comprehensive baseline evaluation is necessary to compare with future measures and provide the patient with accessible and understandable reports. 

Previous studies have shown that health benefits may not be a motivator to maintain physical exercise practice when other objectives are pursued, such as weight loss [61,101]. In this case and as was stated below, it is not recommended to focus on weight loss but on enjoyment and other variables.

One of the most powerful reasons to participate in physical exercise programs is maintaining independence [42] and improving performance in daily activities, such as rising from a chair or climbing stairs [69]. Since changes in the ability to perform activities of daily living are usually slow and progressive, it may be necessary to educate patients and let them see the improvements they are achieving, so the participants will be able to adequately value what exercise can do for them [43].

Self-monitoring through perceived exertion could assist individuals to feel more confident about controlling their intensity level [37]. The use of different devices or apps is another way to monitor physical exercise. In this case, the information is often not about the quality of the execution, but about the quantity (number of steps, kilometers, etc.). These technological devices have the potential to reinforce the behavior, improve social support, and even increase self-efficacy and perceived competence, which will be discussed in the next section.

In sum, perceived benefits are crucial to continuing exercise. The subjective perception of the benefits should be complemented by objective measures that the patient can understand and interpret. Thus, an evaluation before starting the program is necessary to compare with future measures and provide the patient with accessible and understandable reports.

### 4.12. Self-Efficacy and Competence

Self-efficacy is one of the most widely known key factors to improve adherence among any kind of population, as 21 of the included reviews pointed out. It can be defined as the individuals’ belief in their own capacity to undertake a specific task and achieve the desired goal [47]. On the other hand, competence is one of the basic psychological needs and is focused on feelings and beliefs during the action [94]. These variables can be affected by social and contextual events, such as feedback, communication, rewards, etc., but also by challenging tailored tasks. Higher perceived competence is related to higher intrinsic motivation and adherence [114], especially when it is accompanied by a sense of autonomy; thus, participants will be more likely to adopt certain activities when they feel efficacious [94].

Self-efficacy is especially relevant among chronic patients since it reflects the perceptions of the patients about the possibility of control their own life and achieve goals. Chronic patients sometimes perceive their diseases as random and inevitable [36,115], completely out of their control. These feelings may lead to higher levels of helplessness, which refers to an attributional style characterized by explaining negative events and consequences as uncontrollable, unpredictable, and unchangeable [116]. On the other hand, a higher health locus of control [48,117] will reduce helplessness. Patient education is crucial to increase self-efficacy and reduce helplessness, enhancing the knowledge about what they can do and what they can change, and improving the overall health [118,119]. In this regard, the enhancement of self-efficacy has the potential to reduce helplessness and depressive feelings and increase adherence [69,120].

During the exercise practice, self-efficacy can be increased through familiarity with the other participants, the staff, the environment (including the facilities and the materials used), and the procedures [73]. Thus, in the initial steps of the exercise program, the staff should be close to the participant and available to explain and solve any doubt, to ensure the patients have no negative feelings until they get familiarized with all the elements of the program. This could be even more important in patients with cognitive impairment [68]. The use of behavioral graded exercise may also increase self-efficacy by increasing confidence in the capability to exercise [57]. In musculoskeletal pain disorders, graded exercise would initially target to weaker muscles or painful areas and gets increasingly more challenging [53].

In sum, self-efficacy and competence are relevant variables to improve adherence, especially among chronic patients, since self-efficacy is related to the perceptions of controlling their own life and achieving goals. Adequate information and education may increase the self-efficacy levels and, consequently, enhance the knowledge about what they can do and what they can change.

### 4.13. Participant’s Active Role

Sixteen of the reviews identified the facilitator role of self-management, self-control, or self-monitoring, including the completion of self-reported diaries. In this line, eight reviews support the relevance of autonomy, which is one of the basic psychological needs to improve intrinsic motivation [103], and empowerment, which would also suggest that giving the participants an active role in the exercise programs may increase the adherence to exercise. When the aim is to increase the adherence of patients to physical exercise in the long-term and not just to our clinical trial, it is crucial to give patients the tools to continue practicing exercise once the program has ended. 

In medicine, Emanuel and Emanuel [121] aimed to explain the relationship between patient and physician and stated that the patient is empowered when he/she is not spoken as a simple patient but as a person who, after receiving adequate information and education, is able to deliberate and understand what is the best option according to his/her characteristics and preferences [122]. Although giving autonomy is encouraged, it is not always possible. For instance, self-monitoring or self-regulation could be not adequate among people who are in the pre-contemplation or contemplation stages of change according to the transtheoretical model [62,81].

It is known that people who are more educated in health issues are more empowered and those who have higher empowerment are more prone to follow a healthy lifestyle [123]. Therefore, giving the patient adequate education, an active role, and some decision power will impact the chances of adhering to a healthier lifestyle. In this regard, the advances in technology have increased the options to upgrade the role of patients and get them engaged in some tasks such as self-monitoring that puts the program in a patient-centered perspective [63].

Additionally, in some patients like those with cardiovascular disease, the self-monitoring of their heart activity may promote physical activity by increasing the perceived safety and also enable the self-regulation of intensity through the adjustment of the heart rate [46,124].

In sum, giving the patients an active role (through self-monitoring or self-management) in the exercise programs may increase adherence. The use of technology may be recommended for that goal.

### 4.14. Goal Setting

Goal setting was identified as a key factor to enhance adherence to exercise by 12 of the included reviews. In line with the discussion above about self-efficacy, expectations, and perceived benefits, goals must be controllable by the participants. Thus, objectives like losing weight as fast as possible are not adequate and may lead to frustration and abandonment. Although it is known that many patients get involved in physical exercise programs just looking for body mass reduction and that health benefits may not be a motivator for maintaining the physical exercise practice in these patients [61,101], it is recommended to avoid unrealistic and uncontrolled expectations. In order to reduce frustration and enhance motivation, goals must be specific, measurable, achievable, realistic, relevant, and timed [125], and must be regularly re-evaluated [62].

Reviews included in this study showed that goals are more likely to be achieved when they are negotiated and agreed by patients and professionals [60]. Furthermore, signing a contract or agreement including consequences and rewards that would follow the desirable or undesirable pre-defined behavior may also enhance motivation and adherence [57,62,90]. These consensual contracts may increase the adherence to exercise through the enhancement of the individual’s self-efficacy and intention to adopt the desirable behavior, which would predict the change in behavior [126].

In sum, adequate goals should be controlled by the patients, avoiding or reducing the number of goals based on uncontrollable results such as weight loss. Furthermore, those objectives should be agreed on and negotiated by patients and professionals.

### 4.15. Limitations

The current study focuses on the identification of key factors and the potential effects of them in adherence to exercise in patients suffering from chronic diseases. However, the detailed strategies that should be considered in the design of future studies and exercise programs are not reported here and further research is needed to review, identify, and summarize the most suitable strategies for each identified key factor.

In general terms, many of the included investigations have identified a lot of limitations related to the assessment and the concept of adherence [12,14,27,48,51,65,66,74]. Variables like “attendance” or “number of patients who abandoned the trial” are often confused with adherence, which should be interpreted not only as the number of sessions attended or the number of patients abandoning, but as the agreement between the target volume and the real volume achieved [24,26]. This problem is widely reported in the included reviews, suggesting that the heterogeneity in the measures limits the extraction of conclusions. Therefore, although the current study considers adherence in line with the definition above, it must be noted that the key factors identified by the included reviews may be related to attendance or other concepts (drop-outs, patients completing a percentage of sessions, patients abandoning, etc.) used in the included reviews within the concept of adherence.

The current review has some limitations. The first one is related to the search strategy. Although most reviews specifically focused on adherence and aimed to identify key factors for adherence to physical exercise will include the word “adherence” in the title, it is possible that some articles have been omitted due to that search strategy. However, a total of 55 reviews and meta-analyses were included in this review, which involved hundreds of clinical trials and observational studies. Therefore, even assuming that some articles may have been omitted, the included number of studies is enough to extract conclusions and describe the existing evidence about strategies to promote adherence to physical exercise. Another limitation could be related to bias in the data extraction. The information was extracted from the results from the reviews and the interpretation of those results by the authors of the included studies. In order to minimize that bias, two authors independently extracted the information and a third one combined the extracted data. The third limitation is derived from the inclusion of heterogeneous populations. In this regard, our aim was to extract information and provide general conclusions that will be useful for any chronic disease (for instance, self-efficacy or the basic psychological needs are going to be relevant for all patients regardless of their condition), but, of course, the specific disease recommendations must also be considered. Despite all these limitations, the authors of this work honestly believe that the current manuscript offers a condensed and specific review of the most beneficial factors to enhance adherence to exercise in patients with chronic diseases and older adults. 

## 5. Conclusions

The current umbrella review has identified several key factors to improve adherence to exercise in patients with chronic diseases, as well as some practical recommendations for professionals and researchers. The initial evaluation of perceived barriers and facilitators is the most frequently described factors in the included reviews, while other factors such as the design of the exercise program (individualization or program length), social support, self-efficacy, and integration in their daily living (affected by the consideration of participant’s preferences and background and also by the accessibility and flexibility of the program) are supported by more than 20 reviews. Furthermore, the presence of a multidisciplinary team, the supervision during sessions, the use of technology, the participants’ education, the presence of pleasant and unpleasant experiences, the communication and feedback, the monitoring, the participants’ active role, and the goal setting were also identified by at least ten reviews. Given the relevance of adherence to exercise to achieve the expected results and avoid sedentary behavior, all those aspects should be considered when exercise programs are designed.

## Figures and Tables

**Table 1:** Summary of the sample characteristics, type of exercise, and number of reviews identified for each sample and exercise type.

Patients Characteristics		Number of Studies	Type of Exercise Interventions Revised
**Cancer**	Cancer patients or survivors	4	Home-based: 1 review Any: 6 reviews
	Advanced cancer	1	
	Non-small cell lung cancer	1	
	Colorectal cancer	1	
**Cardiovascular disease**	Heart failure	2	Center-based: 1 review Any: 6 reviews
	Under cardiac rehabilitation program	4	
	General cardiovascular conditions	1	
**Older adults**	Falls prevention	3	Center-based: 2 review Home-based: 1 review Any: 5 reviews
	Healthy elderly	5	
**Musculoskeletal disorders**	Low back pain	1	Center-based: 1 review Home-based: 1 review Any: 10 reviews
	Arthritis	3	
	Osteoporosis/osteopenia	1	
	General/multiple musculoskeletal pain or chronic conditions	7	
**Obesity or weight loss**		3	Center-based: 3 review
**Intermittent claudication**		2	Center-based: 1 review Any: 1 review
**Mild cognitive impairment and dementia**		2	Center-based: 1 review Any: 1 review
**Parkinson**		1	Any: 1 review
**Type 2 diabetes**		1	Any: 1 review
**Solid-organ transplant candidates**		1	Any: 1 review
**Under vestibular rehabilitation**		1	Home-based: 1 review
**Different chronic diseases**		6	Center-based: 1 review Home-based: 2 reviews Any: 3 reviews
**Exercise Referral schemes**		4	Center-based: 2 reviews Any: 2 reviews

Center-based: exercise programs specifically conducted in public or private centers; Home-based: exercise programs conducted at home; Any: including both center-based of home-based.

**Table 2:** Summary of key factors according to different conditions.

Key Factors	Sub-Key Factors	Number of Reviews Including Each Key Factor					
		Total	Cancer	CVD	Musculo-Skeletal Disorders	Older Adults	Obesity/Weight Loss
Exercise characteristics design	Characteristics of the exercise are individualized and scientifically correct	23	3	3	4	4	2
	The duration of the exercise intervention is not too long	10	-	1	1	3	1
Multidisciplinarity	Multidisciplinary program	12	-	1	5	2	2
Supervision	Supervision	17	1	1	4	4	1
Technology	Use of adequate technology	12	-	3	1	3	1
Initial exploration of participant’s characteristics, barriers, and facilitators	Previous habits and physical and mental health status of the participants are known	36	7	6	5	2	3
	Barriers and facilitators are explored before the exercise program is delivered to search for alternatives	29	5	6	5	3	2
Participants education, adequate expectations, and knowledge about risks and benefits	Participants are educated about physical exercise in their condition	17	2	4	3	2	1
	Participants are adequately informed about the risks and benefits of the program	15	-	3	1	4	1
	Adequate expectations	15	-	3	3	3	1
Enjoyment and absence of unpleasant experiences	Enjoyment	10	-	1	3	2	1
	Absence of unpleasant experiences	9	1	-	3	1	1
Integration in daily living	Participant’s preferences and background are considered in the program to enhance its integration into their lifestyle	29	3	2	5	5	3
	Good accessibility, adequate place, and flexibility in the schedule	21	4	3	3	4	2
Social support and relatedness	Social support from peers and family	22	1	5	4	3	1
	Social support from the professional	22	2	3	5	2	1
	Relatedness	11	-	2	2	3	-
Communication and feedback	Intra-session feedback	11	2	2	3	2	1
	Bilateral and fluid communication with the staff	16	-	2	3	3	1
Available progress information and monitoring	Objective information for patients to know their progress	17	1	3	2	5	2
	Exercise is monitored	16	2	4	-	3	2
Self-efficacy and competence	Self-efficacy	21	2	3	3	3	2
	Competence	7	-	3	-	1	-
Participant’s active role	Self-management, self-control, and self-monitoring	16	1	3	4	1	-
	Autonomy and empowerment	8	-	1	1	2	-
Goal setting	Objectives are clear and established with the patient	12	1	2	5	-	2

Each number represents the number of reviews that support each factor, overall, and according to the condition for those with 3 or more reviews identified. CVD: cardiovascular disease.

## Data Availability

Not applicable.

## Supplementary Materials

The following are available online at https://www.mdpi.com/1660-4601/18/4/2023/s1, Table S1: Summary of the data collected from the reviews and meta-analysis included.
