# Supplementary material for: Key Factors Associated with Adherence to Physical Exercise in Patients with Chronic Diseases and Older Adults: An Umbrella Review
Source: Int J Environ Res Public Health. 2021 Feb 19;18(4):2023. doi: 10.3390/ijerph18042023 (PMC7922504; doi:10.3390/ijerph18042023)
Supplement: Supplementary file 1 [file ijerph-18-02023-s001.pdf]

Study	Study design	Nºarticles (sample size)	Target population	Selected intervention/s	Aim of the review	Adherence type	Main conclusions
MIXED CHRONIC DISEASES							
Albergoni 2019	Review	8 articles (n=205)	Chronic disease patients reporting fatigue.	Any intervention based on physical activity or physical exercise aimed to improve the adherence by using monitoring technology.	To explore the use of monitoring technology to improve adherence to physical exercise programs in home and/or rehabilitation settings.	A and D	The evidence was not enough to establish whether monitoring technology is effective to increase the adherence to physical exercise in chronic patients. More research is needed to determine whether monitoring technology can enhance adherence and, if so, future studies must provide further evidence-based recommendations.
Bullard 2019	Review and meta-analysis	30 articles (n=3721)	Multiple chronic diseases	Interventions of at least 12 weeks, moderate intensity, and aerobic exercise training.	To evaluate the possible adherence and dropout rates differences between patients practicing aerobic physical activity programs, and between diseases.	B and C	The adherence rate to physical activity interventions was 77% with a drop-out rate of 7%. Thus, people with chronic illness can participate in physical activity programs of 3 and more month of duration, including different levels of supervising and enough level for a health positive effect.
Shore 2019	Review	11 articles	Multiple chronic disease and health conditions patients	Exercise referral schemes (ERS)	To describe rates and participant characteristics associated with ERS, with attendance and with adherence levels in ERS.	B	There was insufficient reporting across studies adherence; and interventions such as ERS, which involves input from various health practitioners, requires robust and consistent recording and reporting of all its facets at every stage of the process.
Emmerson 2019	Review and meta-analysis	14 articles (n=2156)	Patients with different health conditions	Exercise interventions to change the patient's behavior, comparing instructions provided using multimedia approaches.	To evaluate the influence of providing instructions using multimedia on adherence and health compared with written/verbal instructions.	A	Providing instructions using multimedia may enhance adherence to exercise, but more research is needed to conclude whether the mode of exercise instructions affects patient outcomes.
Jansons 2016	Review and meta-analysis	11 articles (n=1231)	Multiple chronic health conditions.	Centre and home-based programs.	To establish which exercise adherence programs are most effective for reaching exercise adherence in chronic health conditions patients who had already participated in a supervised exercise intervention.	B and D	No difference was found about the adherence rates among the four follow-up strategies considered (supervised center programs; home-based exercise interventions with telephone follow-up; home-based programs without follow-up; and weaning programs that transitioned patients to an independent, off-site exercise program)

CANCER							
Sheill 2019	Review	18 articles (n=952)	Advance cancer patients (metastatic or palliative)	Structured exercise training, excluding general physical activity recommendations and low intensity practices.	The global aim was to systematically review the advanced cancer patients' participation in exercise programs and to define the characteristics of exercise interventions in relation with their recruitment and attrition rates.	E	The main recruitment problems were the lack of time (present as the principal cause of drop-out too together with the disease progression status) and complications to travel to the exercise centers. The adherences rates were varied because of the heterogeneity in the adherence definition.
Ormel 2018	Review	15 articles (n=2279)	Cancer patients in any treatment phase	Any exercise intensity intervention, supervised and unsupervised.	To detect the adherence prognosticators to exercise programs in cancer patients, during and after treatments.	E	The main adherence predictors to exercise programs were the rehabilitation center location, former exercise experience, high motivation, and few limitations.<br>Participants with less adherence could have an individualized exercise program and extra assistance.
Driessen 2017	Review	11 articles (n=437)	Non-small cell lung cancer (NSCLC) patients.	Exercise programs of moderate intensity with a duration of 16 weeks	To review the feasibility and effectiveness of articles with pre and re- habilitation interventions (involving home-based), and NSCLC patients by assessing physical fitness, and to explain adherence and therapy tolerance, and recuperation.	B and C	Home-based interventions with significant or clinically relevant increases in physical fitness can reach the highest patients and/ or exercise adherence. The home-made programs that combine resistance and endurance training with supervision and personalization, appears to enhance physical fitness, adherence, treatment tolerance, and recovery.
Beaudry 2015	Review	18 articles (n=1149)	Cancer survivors	Training programs during and after cancer treatment.	To investigate the supervised aerobic exercise training programs efficacy on improving VO2peak in cancer survivors.	B	Adherence may explain the heterogeneity of benefits. The attendance to the sessions could indicate the influence of treatment side-effects and patients' motivation.
McCahon 2015	Review	5 articles (n=1932)	Colorectal adenoma patients	Trials assessing a behavioural intervention to a change in physical activity and/ or diet.	To explore physical activity and diet interventions to which colorectal adenoma patients are more probable to adhere	A, B and D	Two interventions were effective in promoting a change in diet archiving an adherence of 50% to the goals in colorectal adenoma patients. Still not effective interventions were identified to enhance the adherence to physical activity.
Kampshoff 2014	Review	18 articles (n=823)	Cancer survivors before, during	Exercise programs, excluding physical	To determinate the contributing factors of exercise adherence and maintenance.	E	Patients with an exercise history had more exercise adherence. However, inconsistent results were noticed for: age, gender, education, psychological

			and/or after primary cancer treatment.	activity recommendations.			and physiological factors as cardiovascular fitness, body mass index, and before intervention physical activity.
Husebø 2012	Review and meta-analysis	12 articles (n exercise group= 703)	Cancer patients and survivors	Moderate to vigorous intensity exercise.	To examine research findings regarding predictors of adherence to exercise programs in cancer populations.	E	Theory of planned behaviour and the transtheoretical model of behaviour change constructions may be relevant as predictors of adherence in cancer papulation.
CARDIOVASCULAR DISEASE							
Xu 2019	Review and meta-analysis	8 articles (n=506)	Cardiovascular disease patients and eligible for cardiac rehabilitation	Cardiac rehabilitation based on mobile applications.	To compare the effectiveness of cardiac rehabilitation delivered using mobile applications and traditional cardiac rehabilitation	B	Mobile applications might be useful to improve the adherence in cardiac rehabilitation, being 1.4 times higher when using mobile applications *versus* the traditional way.
Marin 2018	Review	10 articles (n=849)	Cardiovascular disease patients (aged >18)	Trials comparing usual care versus the use of an activity monitoring device.	To assess activity monitoring devices or apps adherence to increase physical activity levels in cardiovascular disease adults	E	The evidence of the use of activity monitoring devices was low. Challenges continue to determine the effectiveness to use activity monitoring devices with objective information.
Matata 2017	Review	7 articles (n=2354)	Older adults (> 65 year) ongoing to a cardiac rehabilitation.	Programs focused on improving enrolment and/or adherence to: exercise, education and/or preserving lifestyle modifications.	To evaluate the evidence of studies that aimed to increase participation and/or adherence to cardiac programs with patients of ≥65 years.	B	This review found weak evidence to indicate that programs can improve enrolment or adherence to cardiac rehabilitation therapies for patients aged ≥65 years. So, it is needed more high-quality research to make practice recommendations.
Ruano-Ravina 2016	Review	29 articles (n=345,095)	Cardiac rehabilitation patients (CR) after having a myocardial infarction.	Cardiac rehabilitation programs	To establish which factors, impact to the participation and adherence rates of patients, who have suffered a myocardial infarction, in cardiac rehabilitation programs.	B and C	The participation in CR programs develops a homogeneous pattern in diverse settings. Health professionals ought to: identify patterns predicting low participation, be aware to the recruitment process of patients in CR programs by adjusting their messages, redesigning programs to encourage participation and adherence and improving the accessibility or the use of technologies where it is possible.
Karmali 2014	Review	7 articles (n=880)	Cardiovascular disease patients who are eligible	Any exercise intervention to improve patients'	To evaluate the effects and benefits of interventions to improve patient uptake of, and	A and B	Most of the articles did not find significant effects on the program's adherence

			for cardiac rehabilitation.	adherence to cardiac rehabilitation	adherence to, cardiac rehabilitation		
Tierney 2011	Review	9 articles (3,231)	Heart failure patients (aged>18).	Any intervention to increase exercise/physical activity adherence	To analyze the effectiveness of the used of strategies to impulse exercise adherence in patients with a stable heart failure.	E	Short-term positive results were related to motivational strategies like goal setting, feedback and problem-solving, but how to sustain that physical activity remains unclear.
Tierney 2011	Review	20 articles (n=306)	Heart failure patients (aged>18) and their relatives.	Any qualitative intervention with adherence to exercise or physical activity.	To examine the barriers and enablers to exercise among heart failure people and to detect possible behaviors and beliefs achieved interventions with the purpose of improving activity levels in heart failure people.	E	Iit is not enough for practitioners to simply tell patients to exercise; adopting positive health behaviors is a complex process, influenced by factors internal and external to an individual, such as self-efficacy and outcome expectancies.
			OLDER ADULTS				
Hughes 2019	Review and meta-analysis	20 articles (n=4419)	Community-dwelling older adults (> 65 years)	Exercise aimed to prevent falling	To identify methods to increase adherence to exercise-based interventions for falls prevention in community-dwelling older adults	A, B and D	Multifactorial approaches guided by theory-based and/or evidence-based modifiable factors are recommended to improve adherence. Tele-communication and integration into activities of daily living might be promising interventions.
Valenzuela 2018	Review	22 articles (n=810)	Older people.	Technology-based exercise programs.	Examines the literature regarding older adults` acceptability and adherence to technology-based exercise interventions.	B	Adherence was higher for technology-based interventions than traditional interventions, and may provide a sustainable means of promoting physical activity and preventing falls in older people
Room 2017	Review	11 articles	Older adults (≥ 65 years).	Interventions aiming to improve exercise adherence.	To examine the interventions with the aim of improve exercise adherence in older people, to determine whether they are effective and to detect the behavioural techniques or theory behind these interventions.	E	It is necessary more theoretically derived interventions and robust measures of exercise adherence in older people, for adequately test the efficacy of these interventions.
Farrance 2016	Review	9 articles (n=19,058)	Community dwelling older adults (>60 years)	Community based group exercise interventions of ≥6 month's duration	To assess the opinions and adherence to community-based exercise programs of ≥6 month's duration.	B	The average adherence was almost 70%. More research is needed to ascertain which factors have the strongest influence on adherence. Program design and other aspects of total volume and intensity should be based on older people preferences.

Picorelli 2014	Review	9 articles (n=1,370)	Older people (>65 years old)	Any kind of exercise program.	To evaluate older adults' adherence to exercise programs and to identify factors associated with better adherence	B	The large variability in the measurement of adherence limited the comparison between studies and the extraction of conclusions.
McPhate 2013	Review	18 articles	Older adults (>60 years) with risk of falling.	Supervised exercise group interventions to prevent falls.	To Systematically review adherence (programs-related factors, adherence rates, relationships between adherence and falls prevention) in exercise intervention to prevent falls.	B and C	Three factors are key to improve adherence. Low duration, fixed exercise programs with high level of motivation.
Horne 2012	Review	11 articles (n=530)	Older adults (≥60 years)	Any kind of physical activity or exercise	To identify psychological, social and organizational barriers and facilitators for physical exercise	E	Group norms and support from family, friends and community groups appear to be relevant to increase exercise and physical activity in South Asian older adults.
Simek 2012	Review and meta-analysis	23 articles (n=2031)	Older adults (≥ 60 years).	Home exercise intervention.	To appraise whether adherence to home exercise interventions for the prevention of falls is related to program characteristics and intervention efficacy in older adults.	B	Older adults' adherence to home exercise programs for the prevention of falls, was low and could be affected by specific intervention characteristics. However, there was no relationship between adherence and intervention efficacy
MUSCULOSKELETAL DISORDERS							
McDonald 2019	Review	9 articles (n=658)	Adults with spondylarthritis (SpA)	Exercise or educational programs aimed to increase the participation in exercise.	To evaluate the adherence to prescribed exercise and educational program, as well as to identify the factors influencing adherence in Adults with SpA	B	The level and factors influencing adherence in SpA remain unclear. Potential factors were: supervision, inclusion of education programs, higher disease severity and delay in diagnosis.
Willett 2019	Review	24 articles (n=2366)	Lower limb osteoarthritis patients, diagnosed by self-report of symptoms or imaging.	Physiotherapy intervention with behaviour change techniques (BCTs)	To detect the effectiveness of BCTs within physiotherapy interventions to promote physical activity adherence in patients with lower limb osteoarthritis.	E	BCTs are more effective in the short and long term, not the medium term, but further research is required. The most effective BCTs across time points were behavioural contract and non-specific reward.
Eisele 2018	Review and	22 articles (n=3715)	Chronic Musculoskeletal Conditions	BCTs interventions.	To investigate the effectiveness of BCTs interventions in patients	B and D	Evidence is shown about BCTs interventions are effective to promote medium-term physical activity adherence.

	meta-analysis		patients (>18 years old)		with CMC to increase physical activity adherence.		
Meade 2018	Review	8 articles (n=1,018)	Musculoskeletal pain patients (between 18 and 65 years)	Interventions which used at least one BCT. The program must be based on structured and prescribe exercise.	To determine the effectiveness of content and context used and the role of theory in BCT interventions to promote adherence to prescribed exercise.	E	There was moderate evidence of five BCTs: social support, goal setting, instruction of behaviour, demonstration of behaviour, and behavioural practice/ rehearsal could enhance exercise adherence in people with persistent musculoskeletal pain. Future research should explore the synergies between BCTs and theory was utilized
Nicolson 2017	Review and meta-analysis	9 articles (n=1045)	Older adults with chronic low back pain and/or hip/knee osteoarthritis.	Any form of therapeutic exercise aimed to enhance adherence.	To evaluate whether interventions aimed at increasing adherence to therapeutic exercise improve adherence more than a contextually equivalent control	E	Booster sessions (occasional periodic sessions after the sessions are ended aimed to reinforce progress or identify obstacles) may be useful. Motivational strategies in patients with chronic low back pain and behavioural graded exercise in people with osteoarthritis are also recommended
Rodrigues 2016	Review	54 articles	Osteopenia or osteoporosis patients	Exercise programs.	to analyze studies utilizing exercise interventions in patients with osteoporosis by assessing the mediators and barriers to train as well as the adherence rates.	E	To improve exercise adherence, programs must control the mediators and reduce the barriers to the practice. Procedures to encourage and evaluate programs adherence were weakly export in most articles, and a large heterogeneous in the type of exercise and its dosage was noticed.
Peek 2016	Review	12 articles	Adults patients who were prescribed a self-management strategy by a physiotherapist.	Any intervention to aid patient adherence to a physiotherapist prescribed self-management strategy.	To examine the effectiveness of interventions used to aid patient adherence to all physiotherapist prescribed self-management strategies.	E	Despite studies which used an activity monitor and feedback system, written exercise instructions, behavioural exercise program with booster sessions and goal setting demonstrating positive influence on patient adherence to exercise, there is insufficient data to endorse their use in clinical practice.
Ezzat 2014	Review	19 articles (n=4,408)	Osteoarthritis or rheumatoid arthritis patients	Interventions aimed to improve exercise adherence.	To evaluate the effectiveness of interventions aimed to improve adherence to exercise	E	The evidence for exercise adherence interventions in the arthritis population is low. The definition of adherence is still heterogeneous.
Bishop 2014	Review	42 articles (n= 7,661)	Chronic musculoskeletal pain patients	Interventions (>3 months) to increase adherence to physical activity/exercise.	To explore the effects of contextual and BCT content of control and target interventions in clinical trials.	A and B	Adherence may be influenced by patient-practitioner relationship and contextual variables

Beinart 2013	Review	11 articles (n=1088)	Chronic low back pain patients	Home exercise interventions.	To identify those factors associated with adherence to exercise at home prescribed by a health care practitioner	E	There was moderate evidence for four subfactors: 1) higher health locus of control, 2) supervision, 3) participation in an exercise program, and 4) participation in a general behavior-change program with motivational strategies.
Jordan 2010	Review	42 articles (n=8243)	Patients with chronic pain in the axial skeleton or large peripheral joints.	Interventions that aimed to improve adherence to exercise and physical activity.	To evaluate the effectiveness of interventions that aimed to increase adherence to exercise and also to identify the factors that could be effective in improving adherence.	A, B and D	Exercise type might not be a relevant factor to improve exercise adherence. The potentially relevant factors were: supervision, individualization, refresher or follow-up sessions, the provision of supplementary materials, and that are based on graded activity, include self-management programs and cognitive behavioral techniques. Firm conclusions could not be drawn due to the large inconsistency and heterogeneity.
McLean 2010	Review	5 articles	Musculoskeletal dysfunction patients.	Two types of adherence interventions: cognitive-behavioural (CB) interventions and the use of supporting materials.	To determine strategies used to enhance adherence with musculoskeletal outpatient treatment.	E	Combined interventions could be efficient at increasing adherence with exercise and clinic appointments, but future research could find the barriers in order to create new strategies for adherence.
Jack 2010	Review	20 articles (n=3,870)	Musculoskeletal dysfunction patients.	Therapeutic exercise administered by physical or exercise therapists.	To discover barriers to treatment adherence in patients with mechanical musculoskeletal dysfunctions and propose strategies for diminishing their impact.	E	Physiotherapists should take in account patient's life circumstances, as well as attitudes, beliefs and barriers and create realistic and individualized treatment plans.
OBESITY OR LOSS WEIGHT PROGRAMS							
Burgess 2017	Review	24 articles (n=5,217)	Obesity adults (BMI ≥ 30 kg m^2).	Lifestyle intervention programs.	To identify barriers to behaviour change and predictors of adherence to lifestyle intervention programs in adults with obesity	E	The most prominent predictors of adherence include early weight loss success, lower baseline body mass index (BMI), better baseline mood, being male and older age.
Lemstra 2016	Review and meta-analysis	27 articles (n= 5,576)	People who want to lose weight through	Supervised weight loss programs.	To evaluate adherence to different weight loss interventions and to identify relevant factors	E	Supervising the programs and adding social supports may improve adherence to weight loss interventions.

			exercise and/or diet				
Burgess 2017	Review and meta-analysis	12 articles (n=2,675)	Adults with a body mass index ≥30	A variety of behavioural treatment strategies.	To check if behavioural treatment strategies can enhance adherence to lifestyle intervention programs in adults with obesity.	A and B	Behavioural treatment strategies enhance adherence to lifestyle intervention programs in adults with obesity. The strategies presented should be incorporated into lifestyle intervention, obesity management and weight loss programs with the aim of promoting adherence and engagement.
MILD COGNITIVE IMPAIRMENT AND DEMENTIA							
Vseteckova 2018	Review	9 articles (n= 1084)	Older people with dementia	Group exercise including walking	To evaluate the barriers and facilitators to exercise adherence in older adults with dementia	B and D	Some barriers were identified including biomedical issues, mental and physical wellbeing, relationships and socioeconomic reasons. The facilitators were: biomedical and physical improvements; feelings and confidence enhancement; therapist and group relationships; and activity related reasons.
Van der Wardt 2017	Review	12 articles (n=9,999)	Mild cognitive Impairment or dementia patients	Any kind of physical exercise	to evaluate strategies used in the intervention design of exercise studies to enhance adherence.	E	Pedometers information material (large print, simple descriptions) and exercise logs can increase physical engagement
INTERMITTENT CLAUDICATION							
Lin 2019	Review	84 articles (n=4742)	Patients with intermittent claudication associated to peripheral arterial disease	Any form of structured exercise (>4 weeks and reporting the number of participants that commenced and completed the program).	To compare completion and adherence rates of exercise programs in traditional exercise vs. alternative exercise interventions among patients.	B and D	Non-traditional exercise may be beneficial for patients with IC as adherence and program completion rates are better compared to those for traditional exercise.
Harwood 2016	Review	23 articles (n= 7,517)	Patients diagnosed with intermittent claudication	Any form of (SEP) regardless of the structure, duration, frequency, or intensity of training used.	To establish the rates of recruitment/acceptance and adherence to SEPs, as well as to determine the reasons for poor rates.	B and C	Details of populations screened, reasons for exclusion, and definitions of adequate uptake and adherence were lacking in most reports. Only 1/3 of screened patients was suitable for and willing to undertake SEP. Changes in SEP may be needed to encourage and/or retain participants.
OTHER DISEASES							

Gaikwad 2016	Review	8 articles (n=1,256)	Home-based vestibular rehabilitation patients	Home Exercise Programs.	To determine the most effective strategies to ensure maximum adherence to Home Exercise Programs (HEP) among patients undergoing vestibular rehabilitation.	B, C and D	Strong evidence for 3 major categories of effective HEP adherence strategies: 1) providing patient with written summary of HEP; 2) asking patients to maintain a record of HEP and symptoms; 3) providing tele-rehabilitation (email and/or telephone support) along with in person treatment sessions.
Wallen 2016	Review	11 articles (n=874)	Solid-organ (lung and heart) transplant candidates	Supervised exercise interventions (4 to 26 weeks).	To systematically review the safety to exercise practicing, and the adherence and efficacy of solid-organ transplant candidates.	B	The adherence of heart and lung transplant candidates to exercise is high and also crucial to optimize potential benefits.
Sumlin 2014	Review	27 articles (n=7,266)	Type 2 Diabetes and depression patients	Lifestyle changes.	Synthesize recent research on depression and adherence to dietary and physical activity recommendations in persons with type 2 diabetes.	B	Depression is associated with lower adherence to diabetes self-care, but the few studies of interventions designed to treat depression and improve diet and physical activity adherence have shown inconsistent results.
Allen 2012	Review	53 articles (n=1,940)	Parkinson's Disease (PD) patients	Exercise and/or motor training for patients with PD.	To determine the (1) disease severity and cognitive status (2) duration, supervision, delivery, and location of the interventions (3) adherence and adverse events	B and C	Most studies involved short duration, highly supervised and facility-based nature, and included only cognitively intact participants with mild-to-moderate disease.
EXERCISE REFERRAL SCHEMES							
Eynon 2019	Review	24 articles (n=2,531)	Participants aged 18-80 years old included in Exercise Referral Schemes (ERS).	Interventions involved referring a patient by a health professional to an exercise specialist to receive an individualized exercise program.	To study the psychosocial factors that determine adherence to an ERS.	E	Key psychosocial variables associated with ERS adherence were social support, intrinsic motivation, psychological need satisfaction, and self-efficacy.
Morgan 2019	Review	33 articles (n=9,231)	Inactive adults (≥19 years)	ERS as gym, exercise classes, swimming, and walking.	To check the factors influencing in longer-term participation in physical activity and referral to, attendance at and successful completion from the perspective of ERS.	E	The findings about barriers and facilitators of adherence to ERS were consistent and provide valuable guidelines that researchers should consider to maximise the adherence to a scheme.
Bachmann 2017	Review	14 articles	Participants in home-based	Home-based exercise programs	To identify factors that influence adherence to home-based	E	Therapists should be informed about the barriers affecting their patients. Adherence to home-based

			exercise programs over 18 years.		exercise, and to design recommendations about how to enhance adherence.		exercise could be increased through provision of good support of the patient from family members, friends, and physiotherapists, and by increasing the patients' self-motivation and self-efficacy.
Pavey 2012	Review	20 articles (n=19,089)	General medical diagnosis.	ERS interventions	To identify factors predictive and evaluate the levels of ERS uptake and adherence.	B	To know the variation in uptake and adherence across ERS and how it might affect physical activity outcomes, future studies need to use quantitative and qualitative methods.
A: time exercising; B: attendance (% completed sessions); C: dropouts (% participants abandoning); D: % target goal achieved; E: any							
